# RETRACTED ARTICLE: MiR-151-3p transferred by cancer-associated fibroblast-derived extracellular vesicles promotes osteosarcoma progression through the CHL1/integrin 1β/TGF-β axis

**DOI:** 10.1038/s41417-021-00304-w

**Published:** 2021-03-15

**Authors:** Peng Wang, Changchao Wang, Leyin Zhu, Ping Li, Xiaobo Tang, Jian Wang, Fangyong Hu, Gaoshan Qiao, Cheng Xie, Chengdong Zhu

**Affiliations:** 1 Department of Orthopaedics, The Affiliated Jianhu Hospital of Nantong University, Jianhu People’s Hospital, Nantong, P.R. China; 2 Department of Orthopaedics, Huaian Tumor Hospital & Huaian Hospital of Huaian City, Huaian, P.R. China; 3 Department of Orthopaedics, The People’s Hospital of Yizheng City, The Affiliated Hospital of Yangzhou University, Yizheng, P.R. China; 4 Department of Central Laboratory, Huaian Tumor Hospital & Huaian Hospital of Huaian City, Huaian, P.R. China

**Keywords:** Cancer, Cell biology

The Editor-in-Chief has retracted this article because after publication concerns were raised regarding multiple errors in the description of the methods and reagents, most notably primer sequences in Table 1. The authors did not provide the methods and the references sequences used to design the primers upon request. Furthermore, errors in the language throughout the text are potentially misleading to the reader. The Editor-in-Chief therefore no longer has confidence in the reliability of the work presented. None of the authors have responded to any correspondence from the editor about this retraction.

## Supplementary information

supplementary information

Former article version

