# Supplementary material for: RETRACTED ARTICLE: MiR-151-3p transferred by cancer-associated fibroblast-derived extracellular vesicles promotes osteosarcoma progression through the CHL1/integrin 1β/TGF-β axis
Source: Cancer Gene Ther. 2021 Mar 15;28(12):1390. doi: 10.1038/s41417-021-00304-w (PMC8636259; doi:10.1038/s41417-021-00304-w)
Supplement: Supplementary file 1 — supplementary information [file 41417_2021_304_MOESM1_ESM.docx]

**
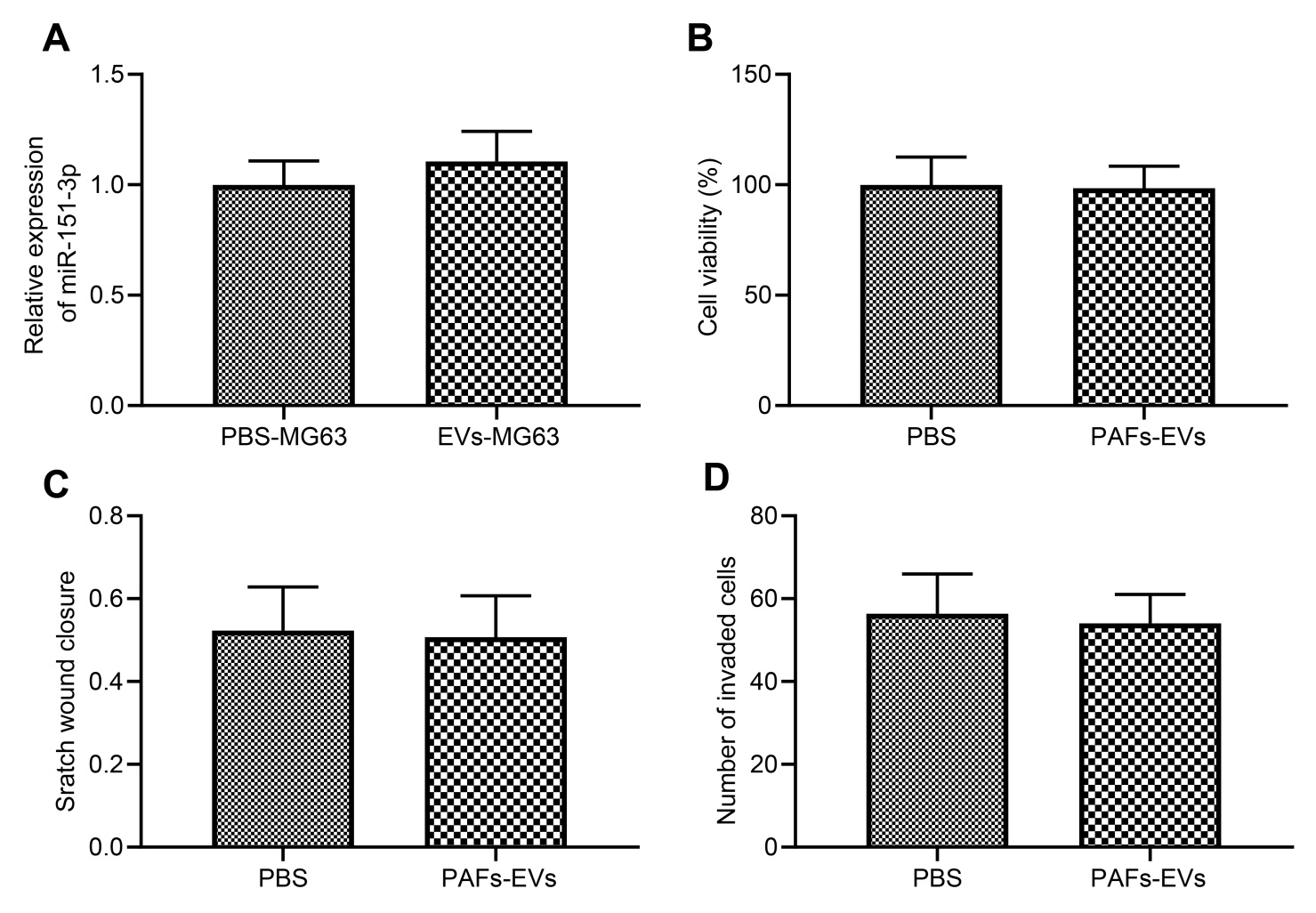
**

**Fig. S1** PAF-EVs have no influence on the migration, invasion and EMT of OS cells. A, Relative expression of miR-151-3p upon treatment with EVs from MG63 cells. B, The viability of MG63 cells treated with PAF-EVs detected by CCK-8 assay. C, The migration of MG63 cells treated with PAF-EVs detected by wound healing experiment. D, The invasion of MG63 cells treated with PAF-EVs detected by Transwell assay.
