# Supplementary material for: RETRACTED ARTICLE: MiR-151-3p transferred by cancer-associated fibroblast-derived extracellular vesicles promotes osteosarcoma progression through the CHL1/integrin 1β/TGF-β axis
Source: Cancer Gene Ther. 2021 Mar 15;28(12):1390. doi: 10.1038/s41417-021-00304-w (PMC8636259; doi:10.1038/s41417-021-00304-w)
Supplement: Supplementary file 2 — Former article version [file 41417_2021_304_MOESM2_ESM.pdf]

Cancer Gene Therapy
https://doi.org/10.1038/s41417-021-00304-w

ARTICLE

MiR-151-3p transferred by cancer-associated fibroblast-derived extracellular vesicles promotes osteosarcoma progression through the CHL1/integrin 1β/TGF-β axis

Peng Wang[1] · Changchao Wang[2] · Leyin Zhu[3] · Ping Li[4] · Xiaobo Tang[1] · Jian Wang[1] · Fangyong Hu[4] · Gaoshan Qiao[3] · Cheng Xie[3] · Chengdong Zhu[3]

Received: 20 May 2020 / Revised: 19 January 2021 / Accepted: 1 February 2021
© The Author(s), under exclusive licence to Springer Nature America, Inc. 2021. This article is published with open access

RETRACTED ARTICLE

Abstract

microRNA-151-3p (miR-151-3p) is a widely reported oncogene with a documented role in tumorigenesis and cancer progression. miRNAs are potent regulators of gene expression that are transferred between cells by extracellular vesicles (EVs). However, there is limited data about the role of EV-derived miR-151-3p in osteosarcoma (OS) progression, or the possible effects of miR-151-3p on the paracrine activity of cancer-associated fibroblasts (CAFs) in OS. To fill this gap of knowledge, we isolated CAF-derived EVs (CAF-EVs) from examined their up-take by human MG63 OS cells and their effects on MG63 biology. We found high expression of miR-151-3p in OS tissues, and miR-151-3p derived from CAF-EVs promoted the process of epithelial–mesenchymal transition (EMT), migration, and invasion of MG63 cells. Notably, bioinformatics analysis, RIP, and dual luciferase report test determined that CHL1 was the direct binding target of miR-151-3p. CAF-EVs mediated the CHL1/integrin 1β axis through miR-151-3p to regulate the TGF-β pathway and then promoted the proliferation, migration, invasion, and EMT of OS cells. Our in vivo results confirmed that EV secretion of miR-151-3p activated the TGF-β pathway through the CHL1/integrin 1β axis to promote the proliferation of OS cells and increase tumor volume and weight. Other effects were to upregulate E-Cadherin and downregulate the expression of N-Cadherin and β-catenin. Hence, our data reveal a potential functional axis mediated by EVs in the tumor microenvironment of OS and suggest potential candidate therapy targets.

These authors contributed equally: Peng Wang, Changchao Wang, Leyin Zhu

Supplementary information The online version contains supplementary material available at https://doi.org/10.1038/s41417-021-00304-w.

✉ Chengdong Zhu
dongdong801208@163.com

[1] Department of Orthopaedics, The Affiliated Jianhu Hospital of Nantong University, Jianhu People's Hospital, Nantong, P.R. China

[2] Department of Orthopaedics, Huaian Tumor Hospital & Huaian Hospital of Huaian City, Huaian, P.R. China

[3] Department of Orthopaedics, The People's Hospital of Yizheng City, The Affiliated Hospital of Yangzhou University, Yizheng, P.R. China

[4] Department of Central Laboratory, Huaian Tumor Hospital & Huaian Hospital of Huaian City, Huaian, P.R. China

Introduction

Osteosarcoma (OS) originates from malignant mesenchymal cells in the bone, which can proceed to develop life-threatening local or distant metastasis, despite optimal management [1]. OS is regarded as the most frequently occurring primary bone sarcoma among children and young adults, but has a bimodal age distribution, with greater incidence in the elderly than among middle-aged people [2]. Moreover, currently available treatment modalities include surgical resection and systemic chemotherapy, giving 5-year event-free survival of patients with localized OS of about 70%, whereas the overall survival rate of patients suffering from metastatic or recurrent OS is below 20% [3]. Therefore, the better understanding of biology and pathogenesis of OS is urgently required to yield novel targeted therapies and to improve oncologic outcomes [4, 5].

In recent years, the tumor microenvironment has attracted research attention regarding its contribution to tumorigenesis, insofar as surrounding fibroblasts are found to be

activated by cancer cells [6]. Of note, cancer-associated fibroblasts (CAFs) can create a favorable environment for invasion and metastasis of cancer cells by interacting with different pathways related to cancer [7]. More importantly, microRNAs (miRNAs) released from extracellular vesicles (EVs) have been recognized as a bridge connecting CAFs, normal fibroblasts, and cancer cells, while dysregulation of miRNAs is closely associated with the formation and function of CAFs [8]. EVs are known to carry diverse molecular cargoes, such as miRNAs, DNA, lipids, and signal peptides [9]. Established roles of EVs include their ability to eliminate unnecessary components for maintaining cellular homeostasis and to mediate intercellular communication [10]. Intriguingly, miRNAs encapsulated in EVs have been reported to possess therapeutic property against malignancy by mediating the metabolism and growth of cells via targeting genes at a post-transcriptional level [11, 12]. Moreover, miR-1228 incorporated in EVs (derived from CAFs in OS) has been demonstrated to exhibit pro-migratory and pro-invasive action by targeting the suppressor of cancer cell invasion (SCAI) [13]. Besides, previously-reported microarray-based analysis has identified miR-151-3p as a putative differentially expressed miRNA in CAF-derived EVs for OS, thus arousing our interest in probing its functional role in regulating the progression and development of OS. Though the functional roles of miR-151-3p in malignancies have been validated in breast cancer and cholangiocarcinoma [14, 15], their implications in OS remain elusive. Moreover, the role of miR-151-3p has been elucidated to depend on its targeting relation with cell adhesion molecule with homology to L1 cell adhesion molecule (L1CAM) (CHL1) [16], which is consistent with prediction results of the preliminary bioinformatics analysis in our study. Given the above-mentioned evidence, we attempted to unravel the role of CAF-derived EV communication in OS involving miR-151-3p and CHL1, aiming to provide a novel insight into developing therapeutic strategies for the management of OS.

Materials and methods

Bioinformatics analysis

The miRNA expression dataset (GSE28423) of OS was retrieved from the Gene Expression Omnibus (GEO) database (https://www.ncbi.nlm.nih.gov/), which included four normal bone samples and 19 OS cell lines. The differentially expressed genes (DEGs) were screened by R limma package with |logFC|> 1 and $p < 0.05$ set as the thresholds. EVs miRNAs data were retrieved from the EV-miRNA database (http://bioinfo.life.hust.edu.cn/EVmiRNA#!/). The intersection between the significant differentially expressed miRNAs in GSE28423, and EVs miRNAs from fibroblasts were obtained to predict the OS-related fibroblasts EVs miRNAs with the jvenn tool (http://jvenn.toulouse.inra.fr/app/example.html). To predict further the downstream target genes of miRNAs, we used three bioinformatics websites, which differed from the binding site matching algorithms, including TargetScan (http://www.targetscan.org/vert_72/), miRDB (http://www.mirdb.org/), and mirDIP (http://ophid.utoronto.ca/mirDIP/). We then obtained the intersection to increase the credibility of the prediction results from jvenn tool. The gene expression levels of OS samples in the TCGA database were obtained through the UALCAN website (http://ualcan.path.uab.edu/) and the predicted target genes were further screened. Finally, the GeneCards database (https://www.genecards.org/) was used to query the downstream regulatory factors of genes, and further predicted the mechanism of action of genes.

The isolation, culture, and identification of CAFs

All fibroblasts were derived from previously operated OS patients. The methods of tissue acquisition and its characteristics have been previously described [13]. CAFs and corresponding para cancer-associated fibroblasts (PAFs) were isolated from OS and the paracancerous tissues digested by collagenase I. Six pairs of matched primary OS adjacent tumor-free tissues (5 cm from the resection margin of the tumor) were obtained. The tissues were immersed in serum-free Dulbecco's modified Eagle's medium (DMEM). Tissues were cut into 1–3 mm fragments and collected into C-tubes supplemented with 5 mL of serum-free DMEM containing 0.5% collagenase I and digested for 1 h. After gentle treatment with MACS dissociator (Miltenyi Biotec, Germany) for 2 min, the single-cell suspension was collected and centrifuged at 1000 rpm/min for 5 min. Cell pellets were suspended with DMEM containing fetal bovine serum (FBS) and seeded into T25 tissue culture flasks. The cells were cultured in high-glucose DMEM (Gibco, Grand Island, NY, USA) supplemented with 10% FBS (Gibco), penicillin (100 IU/mL), and streptomycin (100 μg/mL), at 37 °C with 5% CO_2. Non-adherent cells and tissues were removed by washing twice with phosphate-buffered saline (PBS) after 48 h and the adherent stromal fibroblasts were then cultured for 4–7 days.

The isolated cells above were grown on overslips at 4×10^4 cells/well, and fixed in 4% paraformaldehyde for 30 min. After washing with PBS, the 0.1% Triton X-100 was added to the mixture for 15 min. Then the cells were blocked in 5% BSA for 1 h, and incubated with the primary antibodies against α-SMA, FSP1, and FAP at 4 °C for 12 h. After washing with TBST for three times, the cells were incubated with the fluorescent secondary antibody for 1 h at room temperature in the dark, and then stained with DAPI for 5 min. After washing three times with PBS, the surface markers α-smooth muscle actin (αSMA), fibroblast-activated protein (FAP), and

RETRACTED ARTICLE

fibroblast-specific protein 1 (FSP1) of CAFs and PAFs were detected by the flow cytometry [17].

Extraction and identification of EVs from CAFs and PAFs

The cancer-associated fibroblast extracellular vesicles (CAF-EVs) and para cancer-associated fibroblast extracellular vesicles (PAF-EVs) were isolated from CAFs and PAFs cells, respectively, in the 3rd–5th generation. Before isolation of EVs, the cell medium was replaced with RPMI 1640 containing 0.5% FBS (Sigma-Aldrich), while CAFs and PAFs were then incubated for further 24 h. The cell culture supernatant was collected and centrifuged at 2000×*g* and 4 °C for 20 min to remove cellular debris. Then the EVs were collected after 1-h ultracentrifugation at 4 °C, and 10,000×*g*. The EVs were suspended in medium containing 25 mM 4-(2-hydroxyethyl)piperazine-1-ethanesulfonic acid before ultracentrifugation. Finally, the supernatant was removed and the EVs were resuspended in PBS and stored at −28 °C. The method was used to enrich CAF-EVs and PAF-EVs by multiple rounds of ultracentrifugation.

As reported previously [18], extracted EVs were characterized by transmission electron microscope (TEM) observation, nanoparticle tracking analysis (NTA), and Western blot analysis. In brief, extracted EVs were fixed with 1% glutaraldehyde, applied as a drop on a carbon-coated copper grid, and stained with 1% phosphotungstic acid. The samples were tested using a JEM-2100 TEM (JEOL, Tokyo, Japan). LSM images were recorded using the PARTICLEMEIRIX system. Nanoparticle tracking analysis (NTA) was performed using a NanoSight NS300 system (Malvern Instruments, Malvern, UK). Brownian motion of extracellular vesicles in PBS was recorded and tracked, while size distribution data were generated by applying the Stokes–Einstein equation. The characteristics of extracellular vesicles were identified by the Western blot analysis. For this purpose, the total protein concentration was determined according to the bicinchoninic acid (BCA) protein assay kit (Beyotime Institute of Biotechnology, Shanghai, China), an 10% SDS gel loading buffer was prepared. The mixture of extracted protein and the loading buffer was boiled for 10 min, cooled on ice, centrifuged, and equivalently added into each lane for electrophoresis by a micropipette, and then the gel protein was transferred to nitrocellulose membrane. The membrane was added with the primary antibodies i.e., CD63 (1:2000, ab216130, rabbit antibody, Abcam, Cambridge, UK,), TSG101 (1:10,000, ab125011, rabbit antibody, Abcam), CD81 (1:10,000, ab109201, rabbit antibody, Abcam), and Calnexin (1:100,000, ab92573, rabbit antibody, Abcam). With an addition of the secondary antibody of goat anti-rabbit IgG conjugated with horseradish peroxidase (HRP) (1:5000, Beyotime Institute of Biotechnology, Shanghai, China.), the membrane was shaken and incubated at 37 °C for 1 h. The membrane was immersed in enhanced chemiluminescence (ECL) reaction solution (Pierce, Waltham, MA, USA) for 1 min, and then exposed after removal of the liquid to the chemiluminescence machine (Shanghai Jing Science and Technology Co., Ltd., Shanghai, China). In this experiment, sodium dodecyl sulfate-polyacrylamide gel electrophoresis (SDS-PAGE) was carried out and the antigen-antibody reaction was conducted after the membrane was transferred. The semiquantitative gray analysis was adopted to analyze the bands through the FluorChem FC2 system (Alpha Innotech, San Leandro, CA, USA) and the gray value of each band was calculated.

Cell transfection

Human OS MG63 cells and human embryonic kidney (HEK) 293 cells were obtained from the American Type Culture Collection (ATCC, Manassas, VA, USA). The MG63 cells were cultured in DMEM supplemented with 10% FBS (Gibco), 1% penicillin, and 1% streptomycin.

MG63 cells were seeded into a 6-well plate at 4×10^5 cells/mL. When the cell fusion reached 80%, the cells were transfected according to the instructions of Lipofectamine 2000 reagents (11668-019, Invitrogen, New York, CA, USA). CFAs were transfected and grouped as followed: i) miR-151-3p-inhibitor, ii) NC-inhibitor. MG63 cells were transfected and grouped as followed: i) miR-151-3p-mimic, ii) NC-mimic, i) si-integrin 1β, si-NC; ii) oe-NC, i) oe-CHL1. Moreover, cell transfection lasted for 24 h. si-integrin 1β, oe-CHL1, miR-151-3p-mimic, miR-151-3p-inhibitor, and negative control siRNA (si-NC), negative control mimic (NC-mimic), and negative control inhibitor (NC-inhibitor) were performed by Shanghai Gene Pharma. Transfection of siRNA or miRNA mimics or inhibitors was performed using Lipofectamine 2000 Reagent (Invitrogen) at a final concentration of 100 nM. Each group of cells was cultured for subsequent experiments at 37 °C in 5% CO_2 for 48 h.

Immunofluorescence

Firstly, cells were fixed with 4% paraformaldehyde for 20 min and permeated with the 0.1% Triton X-100 for 5 min. Then, cells were incubated overnight with the specific primary antibodies (1:100) at 4 °C followed by incubation with the fluorescent secondary antibody (Life Technologies, USA). The nuclei were stained with DAPI (100 ng/mL, Roche, USA). All labeled cells were examined with a Leica confocal fluorescence imaging microscope and LAS AF version 2.0 software (Leica Microsystems, Germany). The specific primary antibodies included Patient-derived α-SMA (ab5694, Abcam), vimentin (ab20346, Abcam), FAP (ab28244, Abcam).

RETRACTED ARTICLE

Table 1 Primer sequence.

Target	Primer sequence (5′−3′)
miR-151-3p	F: GGATGCTAGACTGAAGCTCCT<br>R: CAGTGCGTGTCGTGGAGT
CHL1	F: ATGATGGAATTGCCATTATGT<br>R: TGGCGGCCGGCCGCTTCATGCCCGGAGTGGGAA
Integrin 1β	F: CTTTGCGTTGTCAGCATGGG<br>R: ACACTGCCATCTGCCTTTCT
U6	F: CGATACAGAGAAGATTAGCATGGC<br>R: AACGCTTCACGAATTTGCGT
GAPDH	F: ACCCAGAAGACTGTGGATGG<br>R: CACATTGGGGGTAGGAACAC

Real-time qPCR

Total RNA (500 ng) was extracted using the Takara Prime Script RT master mix kit (Takara, No. RR037B) according to the manufacturer's instructions. The RT-qPCR was performed using the SYBR Premix Ex Taq II kit (Takara, No.2) in a 20 μL reaction mixture containing equal amounts of cDNA. miRNAs were reversely transcribed and relatively quantified using the TaqMan MicroRNA Reverse Transcription Kit (Thermo Fisher Scientific, 4366596). All mRNA primers used in this study were in accessory material (Table 1). TaqMan® Probes were purchased from Thermo Fisher Scientific (miR-151-3p, Thermo Fisher Scientific, No.1). β-actin was used as an internal reference gene for mRNA and U6 for microRNA, because they are stably expressed in cells and have been widely used for determining gene expression. The calculation of each sample is based on their relative horizontal threshold period (Ct) value normalized Ct value internal reference gene using formula $2^{-\Delta\Delta Ct}$ (ΔCt = Ct target gene-Ct β-actin/U6).

Laser confocal microscopy

After CAF-EVs were extracted, they were stained with PKH67. According to kits instructions (pkh67gl-1kt, Sigma-Aldrich, St. Louis, USA), EVs were mixed with Diluent C, and PKH67-Diluent C dye was then added rapidly, mixed, and incubated for 5 min. Then staining was terminated by adding 2 mL 10% BSA in PBS (D8537). The liquid was transferred to the bottom of the tube slowly and carefully, added with 1.5 mL sucrose solution, and ultra-centrifuged at 190,000 r/min for 2 h at 2–8 °C. The media and interface layers were carefully aspirated and the extra-cellular vesicle pellet was resuspended in PBS by gently blowing. Thereafter, it was transferred to the Amicon centrifugal filter and centrifuged at 3000 r/min for 40 min in a high-speed centrifuge to reduce the volume to 0.5–1/mL.

MG63 cells were routinely cultured, plated, and replaced with fresh medium after 48 h. Next, the cells were stained with DiL to become red and co-cultured with 0.05 μg PKH67-labeled EVs per 10^3 cells [19] or PBS for 24 h [20]. After washing three times with PBS, the cells were fixed with 4% paraformaldehyde at room temperature for 30 min. Then, the cells were washed three times with PBS and stained with DAPI at a density of 100 ng/mL (36308ES11, Yisheng Bio, Shanghai, China) for 5 min. The images were observed and captured with a laser confocal microscope (DMi8, Leica, Wetzlar, Germany). There were two groups: (1) PBS group (MG63 cells were added with PBS culture only), (2) CAF-EVs group (MG63 cells were co-cultured with PKH67 labeled CAF-EVs).

Dual-luciferase reporter assay

The target of miR-151-3p was verified to be CHL1 by luciferase reporter assay while the 3′UTR of miR-151-3p and CHL1 were artificially synthesized. The gene promoter fragments were introduced into the ARE-Luc reporter gene using the endonuclease sites *Nhe I* and *Bgl II* (Shanghai Yisheng Biotechnology Co., Ltd.), whereas the complementary sequence mutation site of the seed sequence was designed on miR-151-3p and wild-type (WT) CHL1. After restriction enzyme digestion, the target fragment was inserted into the pGL3 reporter plasmid using T4 DNA ligase. The correctly sequenced luciferase reporter plasmids (pGL3-miR-151-3p WT) (pGL3-CHL1 WT) and (pGL3-miR-151-3p MUT) (pGL3-CHL1 MUT) and (pGL3-Control) were co-transfected into HEK293T cells (purchased from ATCC) with miR-151-3p-mimic and NC-mimic, respectively. After 12 h of transfection, the cells in different groups were treated with DMSO for 24 h. Before being lysed by the buffer of double-luciferase reporter assay kit (E1910, Promega), the cells were washed with 1× PBS. Afterward, the luciferase activity was measured with the Glomax 20/20 luminometer fluorescence detector (Promega). Each group of experiments was repeated three times.

CCK8 assay

Cell viability was analyzed using Cell Counting Kit 8 (CCK-8, Dojindo, Kyushu Island, Japan). After being treated with EVs, MG63 cells were seeded into 96-well

RETRACTED ARTICLE

plates with 5×10^3 cells/well for 24 h. After that, each well was added with 10 μL of CCK-8 solution and incubated for 2 h at 37 °C. Absorbance was measured with a molecular device (LLC, Sunnyvale, CA, USA) at 450 nm. The experiment was repeated three times.

Wound healing assay

MG63 cells (2×10^5 cells/well) were seeded in a 6-well plate for 24 h. Then 100 μg/mL EVs were added into fresh medium (the number of EVs is the same as Transwell analysis). After 48 h of incubation with EVs, the cell monolayer was scraped with a 1 mL pipette tip. Then, the cells were cultured in a serum-free medium, and the migration ability was evaluated under a bright-field microscope at 0 and 48 h.

Transwell assay

Before the experiment, 50 μL Matrigel Matrix (Sigma, USA) was added to the chamber to coat the filter membrane. Next, 200 μL cell suspension (2×10^5 MG63 cells/well) was seeded in the upper chamber. The remaining steps were the same as above. The number of stained cells was counted under an inverted microscope (XDS-800D, Shanghai Caikang Optical Instruments Co., Ltd., China) and was expressed as the mean.

RNA-binding protein immunoprecipitation (RIP) assay

The binding of miR-151-3p to Argonaute2 (AGO2) protein was detected using the Magna RIP RNA-Binding Protein Immunoprecipitation kit (Millipore Billerica, MA, USA). Cells were lysed using an equal volume of RIPA lysis buffer (P0013B, Beyotime Biotechnology Co., Shanghai, China) on an ice bath for 5 min and centrifuged at 14,000 rpm at 4 °C for 10 min for isolation of the supernatant. A part of the cell extract was used as input, while the other part was incubated with the antibody for co-precipitation. The steps were as follows: In each co-precipitation reaction system, 50 μL of magnetic beads were washed and re-suspended in 100 μL of RIP Wash Buffer (EHJ-BVIS08102 Xiamen Jiahui Biotechnology Co., Ltd., China). Each group was added with 5 μg of antibodies for binding. The magnetic bead-antibody complexes were washed and resuspended in 900 μL of RIP wash buffer, followed by addition of 100 μL of cell extract to incubate at 4 °C overnight. The sample was placed on a magnetic pedestal to collect the magnetic bead-protein complexes. The samples and input were digested by protease K, respectively. Then, RNA was extracted for subsequent PCR detection. RNA was extracted from exosomes using the Total Exosome RNA Isolation Kit (Invitrogen, Carlsbad, CA, USA). The extracted RNA was subsequently used for the RT-qPCR assay. Total RNA was extracted using the TRIzol reagent (Invitrogen, Carlsbad, CA, USA). Besides, the antibody used for RIP was AGO2 (ab32381, 1:50, Abcam) mixed at room temperature for 30 min, and IgG (1:100, ab109489, Abcam, UK) as a negative control.

Western blot analysis

Total cellular proteins were extracted using RIPA lysis buffer according to the instructions (R0010, Solarbio). The cells or tissues were lysed at 4 °C for 15 min followed by centrifugation at 15,000 rpm. The protein concentration of each sample was determined by the BCA kit (20201ES76, YEASEN Biotechnology Co., Ltd., Shanghai, China). Quantification was performed based on concentrations while proteins were separated by 10% sodium dodecyl sulfate-polyacrylamide gel electrophoresis (SDS-PAGE) first at 80 V for 35 min and then 120 V for 45 min. Then the protein was transferred onto polyvinylidene fluoride (PVDF) membranes (Amersham, Massachusetts, Boston, USA) in the transfer buffer under the condition of 350 mA for 90 min. The membranes were blocked with 5% nonfat dry milk for 1 h at the room temperature. Diluting rabbit primary antibodies: CHL1 (1:1000, 2524), integrin 1β (1:1000, 2171), N-Cadherin (1:2000, ab20346, Abcam), β-catenin (1:2000, ab32572, Abcam), E-Cadherin (1:2000, ab1416, Abcam), FAP (1:2000, ab28244, Abcam), α-SMA (1:2000, ab5694, Abcam), FSP-1 (1:2000, ab124805, Abcam), and GAPDH (1:10000, ab181602, Abcam) were added for incubation at 4 °C overnight. The membranes were washed for three times with TBST (each for 5 min). The dilutions of HRP Goat Anti-Rabbit IgG Antibody (ab205718, 1:20,000, abcam, Cambridge, UK) were added for 1 h at room temperature. After three washes of 5 min each with TBST, the developer solution was added for imaging. Gray values of bands were analyzed by ImageJ (version 1.48, National Institutes of Health). The experiment was repeated three times.

Establishment of subcutaneous tumor xenografts model in mice

Male Balb/c nude mice ($n = 40$, 4–6 weeks, 15–20 g, J004, Nanjing Junke Biological Engineering Co., Ltd., China) were randomly divided into four groups. The clean laminar airflow frame and room in the feed barrier system were regularly exposed to UV rays while cages, bedding, drinking water, and feed were autoclaved and sterilized at room temperature of 24–26 °C and relative humidity was maintained at 40–60% in the animal quarters. The study was approved and reviewed by the Clinical Ethics Committee of The People's Hospital of Yizheng City, The Affiliated Hospital of Yangzhou University. CAFs were digested, re-suspended, and then centrifuged multiple times to remove FBS as possible from suspension. Two hundred

RETRACTED ARTICLE

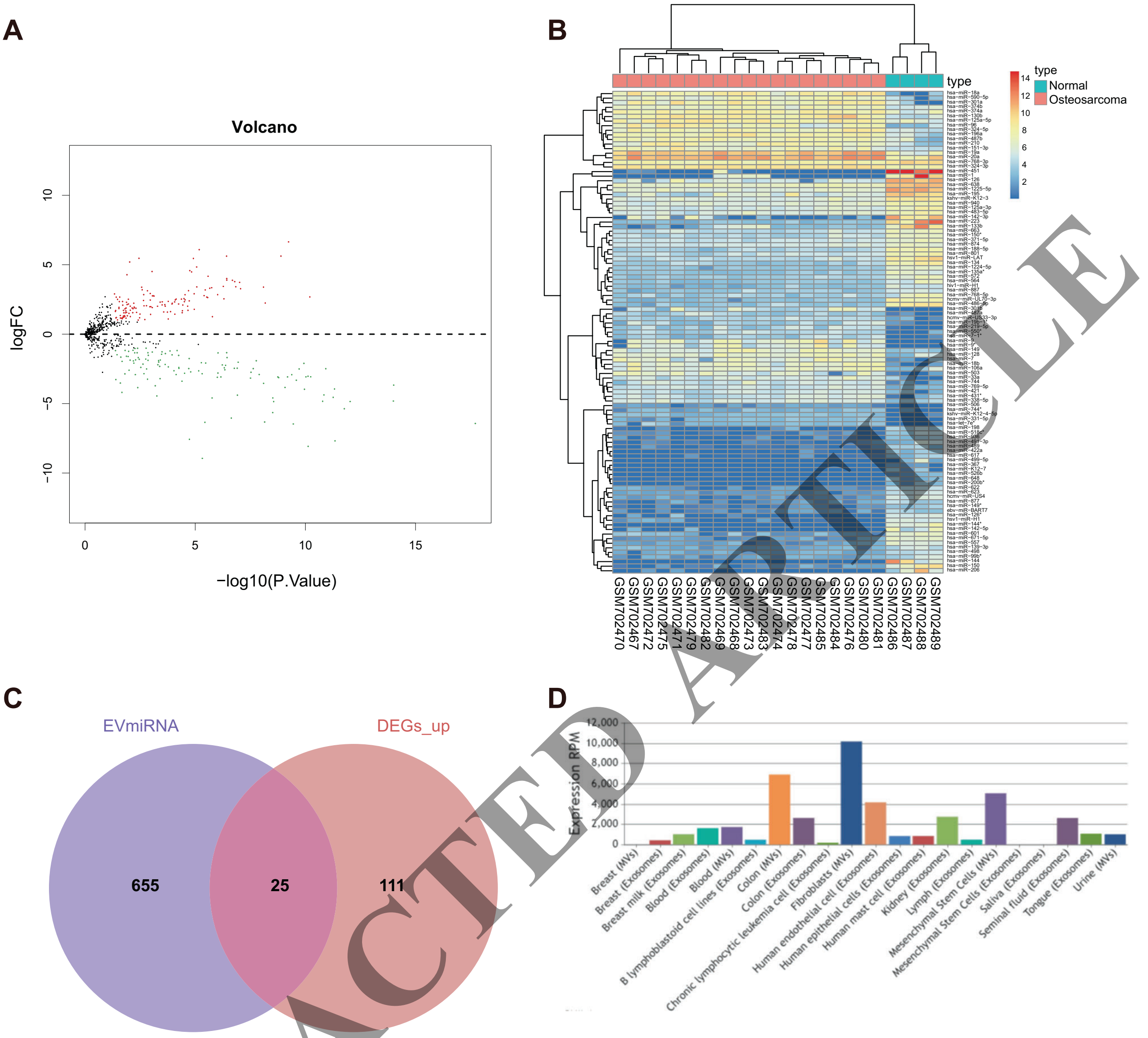

Fig. 1 Bioinformatics analysis. A Volcano graph: abscissa indicates $\log_{10}$ *p*-values and ordinate axis indicates logFC; each point represents a gene, the red dots indicate upregulated genes and the green dots indicate downregulated genes in the OS sample genes. **B** Heatmap of the top 105 genes in OS-related miRNA expression dataset GSE28423. **C** Venn map of the intersection of the GSE28423 high differentially expressed miRNA and fibroblast EVs miRNAs. **D** The mean expression of miR-151-3p from various EVs in the EVmiRNA database.

RETRACTED ARTICLE

microliter of cell suspension at 5 × 10^6 cells/mL were injected into the right shoulder blades of each nude mouse slowly. The next day, miR-151-3p mimic or miR-151-3p inhibitor, and their negative controls (100 nM) were injected into the tail vein. The mice were observed and injected every two days. After 4 weeks, mice were sacrificed by overdose with 9% pentobarbital sodium (P3761, SIGMA, St. Louis, USA) and the tumors were isolated. During treatment, the shortest diameter (a) and longest diameter (b) of the tumors were measured weekly with Vernier calipers; the tumor volume was calculated according to the formula $\pi(a^2b)/6$, and the tumor mass was weighed with a balance. Peripheral blood was collected when the mice were sacrificed and centrifuged to prepare serum. EVs in the serum were extracted using the above method and then their miR-151-3p expression was detected by RT-qPCR analysis. The tumor tissues were fixed in 10% formaldehyde and the routinely dehydrated and embedded for later examination.

Statistical analysis

GraphPad Prism 8.0 (GraphPad Software, La Jolla, CA, USA) was used for statistical analysis and all experiments were repeated at least three times. All data conform to normal

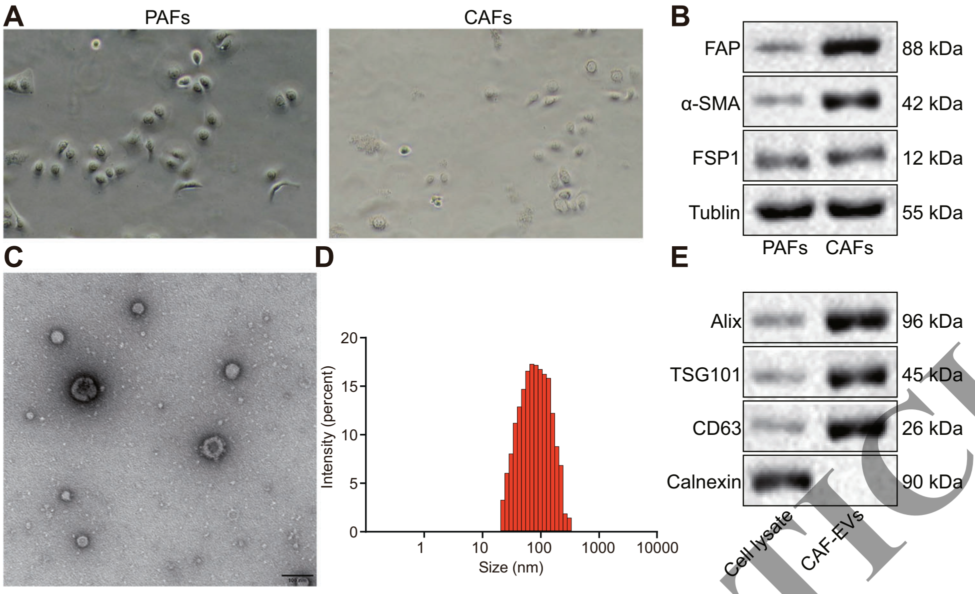

Fig. 2 Morphology of CAFs and identification of EVs. A Morphological characteristics of PAFs and CAFs under an optical microscope (scale bar = 100 μm). **B** Western blot analysis of the expression of CAF markers (FAP, FSP1, and α-SMA) in CAFs and PAFs. **C** Morphological characteristics of CAF-EVs and PAF-EVs under a TEM. (scale bar = 100 nm). **D** The size distribution of CAF-EVs and PAF-EVs measured by NTA. **E** Western blot analysis of the expression of EV specific surface marker proteins CD63, TSG101, Alix and Calnexin in CAF-EVs and cell lysate. (*$p < 0.05$, the data results are measurement reported as mean ± SD, with analysis by one-way ANOVA with Tukey's post hoc correction. The experiment was repeated in triplicate.

RETRACTED ARTICLE

distribution and homogeneous variance test. Measurement data were presented as the mean ± standard deviation (SD). Comparisons between the two groups were conducted with an independent sample *t*-test, while comparisons among multiple groups were performed with one-way ANOVA analysis of variance. Tukey's test was used for posthoc testing. $p < 0.05$ was considered statistically significant.

Results

High expression of miR-151-3p in OS

Through analysis of the OS miRNA expression dataset GSE28423, we screened out 258 differential genes, among which 136 genes were upregulated and 122 genes were downregulated (Fig. 1A). Then top 105 genes were selected to draw the expression heatmaps (Fig. 1B). The 680 miRNAs of fibroblast EVs were obtained from the EVmiRNA database. Then, 25 miRNAs (the intersection of the highly expressed differential miRNAs of dataset GSE28423 and the fibroblast EVs miRNAs) were obtained by jvenn (Fig. 1C). Moreover, the EVmiRNA database showed that miR-151-3p had the highest mean expression in fibroblast EVs (Fig. 1D), while the heatmap of GSE28423 differential gene expression showed that miR-151-3p was highly expressed in OS. Therefore, miR-151-3p was selected as the objective gene for the subsequent in vivo and in vitro studies.

Release and identification of CAF-EVs and PAF-EVs

The main CAFs and corresponding PAFs from OS tumor tissues were isolated by treatment with 0.5% type I collagenase and the cell's morphology and their growth were observed under an inverted microscope. Our results exhibited the slow growth of PAFs, which formed a flat fusiform shape, had a flat oval sac-like nucleus, significantly visible nuclear cuts, and abundant cytoplasm. On the other hand, CAFs grew rapidly, with a long fusiform or polygonal shape, a slightly wider medial aspect mainly protruding at both ends, blurred outline, and an irregular arrangement (Fig. 2A). For further analysis of CAFs and PAFs, the surface markers FAP, α-SMA, and FSP1 were tested by Western blot analysis. We found that the expressions of CAFs-specific markers FAP, FSP1, and α-SMA were higher than that of PAFs (Fig. 2B).

After sequential centrifugation to obtain the supernatant of CAFs, CAF-EVs, and PAF-EVs were extracted by ultracentrifugation. TEM results showed that CAF-EVs exhibited typical morphological characteristics of EVs like cup-shaped or spherical (Fig. 2C). NTA analysis found that the size of CAF-EVs ranged from 40 to 200 nm and the mean size was 108 nm (Fig. 2D). Western blot analysis demonstrated positive expression for EV-specific surface markers CD63, TSG101, Alix, and negative expression for Calnexin. In contrast, in cell lysates there was poor expression for CD63, TSG101,

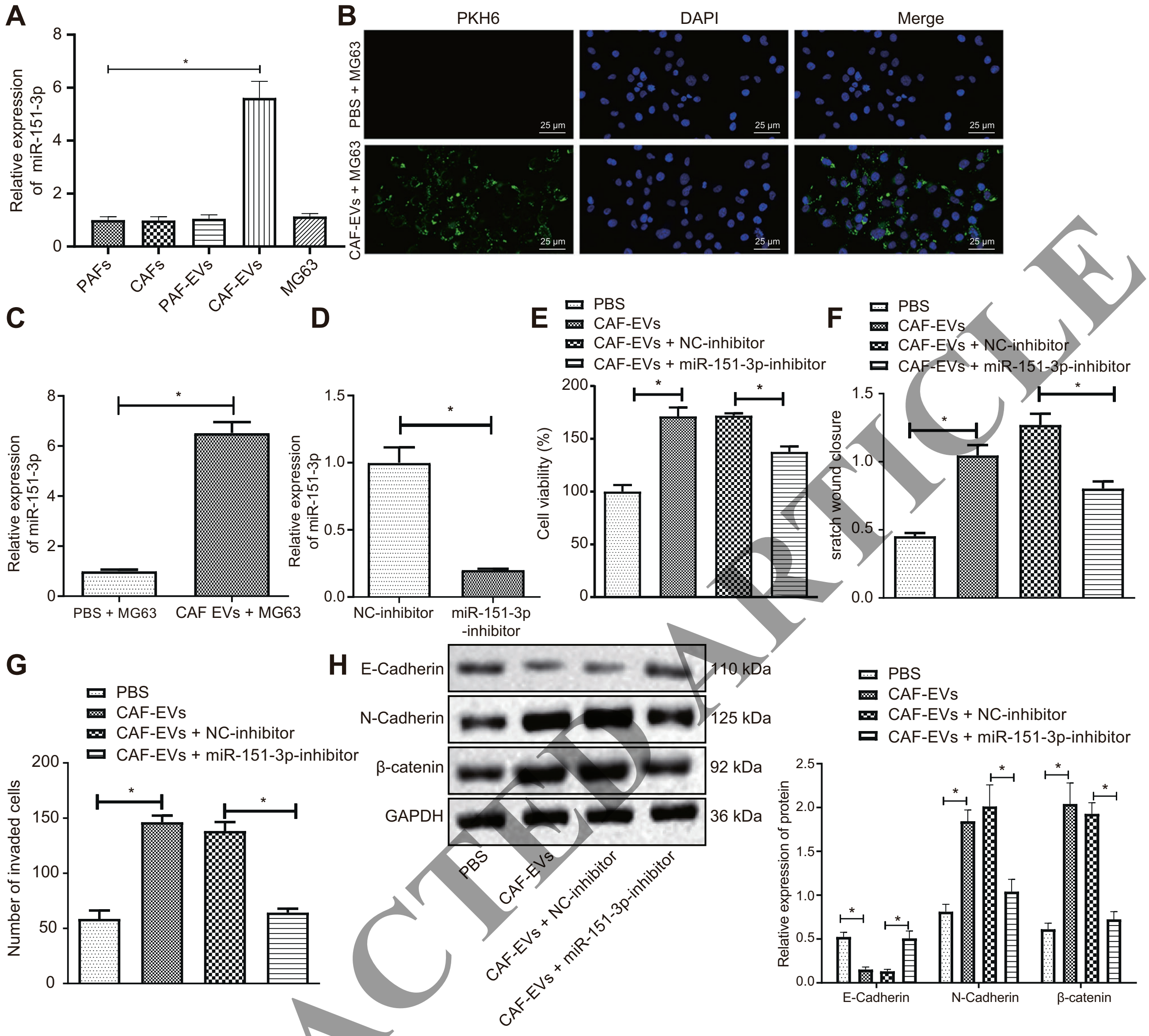

Fig. 3 MiR-151-3p derived from CAF-EVs promotes EMT, migration, and invasion of OS cells. **A** qPCR detected the expression of miR-151-3p in different treatment groups (PAF, CAF, PAF-EVs, CAF-EVs) and MG63 cells. **B** A confocal laser microscope observed the phagocytosis of CAF-EVs or PBS-treated MG63 cells (scale bar = 400 μm). Blue: the nucleus was stained with DAPI. Green: PKH67 fluorescence signal. **C** qPCR detected the miR-151-3p expression in PBS-MG63 or EVs-MG63 cells. **D** qPCR detected the expression of miR-151-3p in NC-inhibitor or miR-151-3p-inhibitor transfected cells. **E** CCK-8 detected the cell viability of MG63 cells after treated with PBS, EVs, EVs + NC-inhibitor and EVs + miR-151-3p-inhibitor, respectively. **F** Cell wound healing experiment to detect the effect of EVs, EVs + NC-inhibitor, EVs + miR-151-3p-inhibitor on MG63 cell migration, respectively. The right histogram is a quantitative analysis of wound closure. **G** Transwell detects the effect of EVs, EVs + NC-inhibitor, EVs + miR-151-3p-inhibitor on MG63 cell invasion. The right histogram is a quantitative analysis of invading cells. **H** Western blot analysis assay detected the expression of EMT-related biomarkers E-Cadherin, β-catenin, and N-Cadherin. GAPDH as a reference for the expression detection. (*$p < 0.05$, the data results are measurement data reported as mean ± SD with analysis by one-way ANOVA with Tukey's posthoc correction. The experiment was repeated in triplicate.

RETRACTED ARTICLE

Alix, but abundant expression for Calnexin (Fig. 2E). According to their characterization, size, and expression of biomarkers it was confirmed that EVs were successfully isolated from the conditioned medium [21]. Furthermore, the EVs extracted from PAF-EVs had the above typical characteristics.

CAF-EVs miR-151-3p promoted EMT, migration, and invasion of OS cells

First, the expressions of miR-151-3p in PAF, CAF, PAF-EVs, CAF-EVs, and MG63 cells were detected by qPCR, showing that the expression of miR-151-3p in CAF-EVs

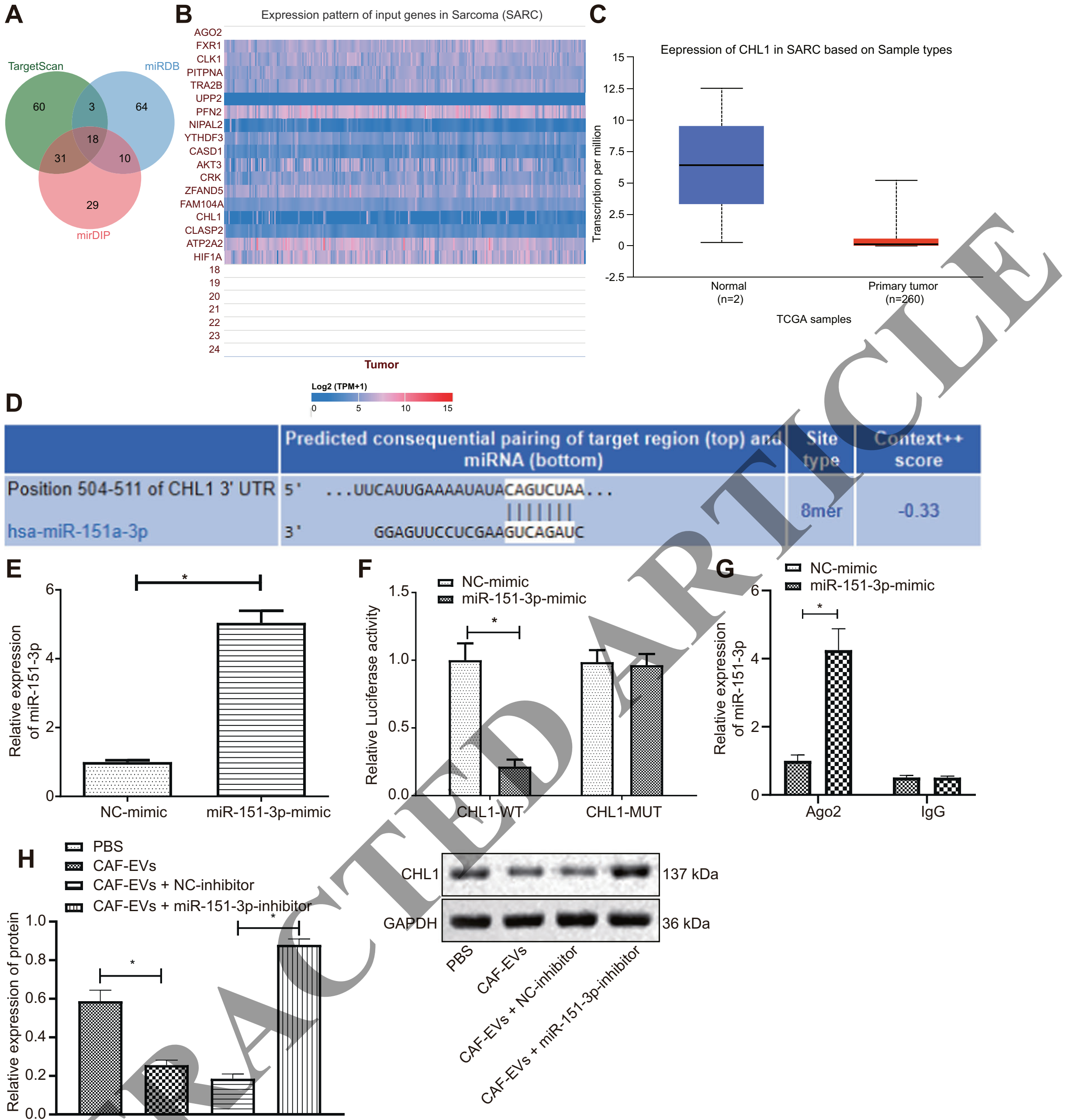

RETRACTED ARTICLE

Fig. 4 CHL1 is a direct target gene of miR-151-3p. A Venn diagram of the intersection of biogenetics website TargetScan, miRDB, and mirDIP predicting the target genes of miR-151-3p. **B** Heatmap of the expression of 18 genes on the UALGA website in OS samples of the TCGA database. **C** Box diagram of CHL1 expression in OS in TCGA data. **D** The binding site of CHL1 and miR-151-3p on the TargetScan website. **E** qPCR detected the expression of miR-151-3p in the miR-151-3p-mimic transfected group. **F** After NC-mimic and miR-151-3p-mimic transfected into CAFs cells, EVs were extracted. Then the EVs and CHL1 3′-UTR wild/mutant dual-luciferase were co-transfected into HEK-293T cells, respectively. The dual-luciferase report detected the binding of miR-151-3p to CHL1. **G** RIP experiment proved that CHL1 was the target of miR-151-3p. **H** After transfected with miR-151-3p-inhibitor and NC-inhibitor, EVs were extracted to treat with MG63 cells, and then Western blot analysis was used to detect the expression of CHL1. (U6 is the internal reference for miR-151-3p expression and GAPDH is the internal reference for CHL1 expression. *$p < 0.05$. The data result is measurement data, mean ± SD indicates that the comparison between the two groups is analyzed by independent sample *t*-test. The experiment was repeated in triplicate.

was significantly upregulated (Fig. 3A). The phagocytosis of CAF-EVs by MG63 cells was detected after the extracted CAF-EVs were co-incubated with MG63 cells. In CAF-EVs-MG63 co-incubation group, the EVs labeled with PKH67 showed green fluorescence and main labeling in the cytoplasm while there was no green fluorescence in PBS-MG63 group, which indicated that CAF-EVs were successfully endocytosed into cells (Fig. 3B). Besides, the

qPCR analysis showed that, compared to the PBS-MG63 group, the expression of miR-151-3p in CAF-EVs-treated MG63 cells was also significantly increased (Fig. 3C).

To confirm the effect of miR-151-3p derived from CAF-EVs on the migration, invasion, and EMT of OS cells, the CAFs cells were transfected with NC-inhibitor and miR-151-3p-inhibitor. The qPCR analysis showed that, compared to the NC-inhibitor, the expression of miR-151-3p was effectively suppressed in the miR-151-3p-inhibitor group (Fig. 3D). Afterward, EVs were extracted from CAFs transfected with NC-inhibitor and miR-151-3p-inhibitor were used to treat with MG63 cells, while CCK-8 was used to detect the cell viability of MG63 cells. Being distinct from the PBS group, the cell viability of the CAF-EVs group was significantly increased, whereas, the cell viability of CAF-EVs + miR-151-3p-inhibitor group was inhibited (Fig. 3E) compared to the CAF-EVs + NC-inhibitor group. In wound healing and Transwell experiments, the migration and invasion of MG63 cells in the CAF-EVs group was higher than that of PBS, while the migration and invasion in CAF-EVs + miR-151-3p-inhibitor group were inhibited (Fig. 3F, G), compared to CAF-EVs + NC-inhibitor group. Next, the expression of EMT-related biomarkers was detected by Western blot analysis. Compared to PBS group, the expression of N-Cadherin and β-catenin was upregulated while that of E-Cadherin was suppressed in the CAF-EVs group (Fig. 3H). Besides, compared to the CAF-EVs + NC-inhibitor group, the expression of N-Cadherin and β-catenin was downregulated and E-Cadherin expression was upregulated in the CAF-EVs + miR-151-3p-inhibitor group. In addition, as shown in Fig. S1, there was no similar result trend in PAF-EVs, suggesting that PAF-EVs had no obvious effect on the migration, invasion and EMT of OS cells. These results suggested that the miR-151-3p derived from CAF-EVs promoted the EMT, migration, and invasion of OS cells.

MiR-151-3p bound to CHL1 3′UTR

To investigate further the role of miR-151-3p in the molecular etiology of OS, we searched for its targets. Previous research had indicated that CHL1 could bind with miR-151-3p to promote the development of OS [22]. Bioinformatics prediction revealed 112, 95, and 88 genes targeted by miR-151-3p from TargetScan, miRDB, and mirDIP, respectively, among which18 common target genes stood out (Fig. 4A). Further analysis of expressions of genes in the OS samples of the TCGA database through the UALCAN website (Fig. 4B) exhibited low expressions of UPP2, NIPAL2, and CHL1, in contrast to the higher expression trend of miR-151-3p. Previously reported studies have indicated CHL1 to be an important tumor suppressor possessing anti-proliferative and anti-metastatic properties [23]. Moreover, the TCGA database showed a low expression of CHL1 in OS (Fig. 4C). Furthermore, the TargetScan website was employed to obtain the 504–511 position on the 3′ UTR of CHL1 and the targeted binding site of miR-151-3p (Fig. 4D). The predicting binding site was detected by the dual-luciferase reporter test. After transfection of miR-151-3p-mimic and NC-mimic into HEK-293T cells, qPCR assay was used to detect the transfer efficiency of miR-151-3p-mimic. The expression of miR-151-3p was significantly upregulated in miR-151-3p-mimic group (Fig. 4E). Compared with the NC-mimic + CHL1WT co-transfection group, the fluorescence intensity of miR-151-3p-mimic + CHL1WT co-transfection group was markedly reduced. However, there was no corresponding change observed in the miR-151-3p + CHL1MUT co-transfection group (Fig. 4F). The abundance of AGO2 binding miRNAs is negatively associated with the inhibitory potential of their target gene. Accordingly, our results from the RIP assay demonstrated that more miR-151-3p was immunoprecipitated upon applying AGO2-specific antibodies relative to the IgG control in miR-151-3p mimic-transfected MG63 cells, suggesting that miR-151-3p could indeed bind with CHL1 (Fig. 4G). After transfection with miR-151-3p-inhibitor and NC-inhibitor, MG63 cells were treated with extracted EVs. Western blot analysis showed, that compared to the PBS group, the expression of CHL1 in EVs group was significantly downregulated, while compared to the EVs + NC-inhibitor group, the expression of CHL1 in EVs + miR-151-3p-inhibitor group was upregulated (Fig. 4H). Collectively, the above-described results of the bioinformatics analysis, RIP, and dual luciferase report test suggest that CHL1 was a direct binding target of miR-151-3p.

MiR-151-3p delivered by CAF-EVs promoted activation of the TGF-β pathway via CHL1/integrin1β axis in vitro to promote OS occurrence

Further analysis by the GeneCards database revealed that integrin 1β is a key interaction factor of CHL1 (Fig. 5A). Existing literature showed that the interaction between CHL1 and integer 1β inhibited OS tumor growth and metastasis [24], while overexpression of integer 1β promoted proliferation, invasion, and migration of tumor cells [22]. Intriguingly, it has been demonstrated that integrin 1β promoted the activation of the TGF-β signaling pathway [25], which could promote the growth and metastasis of OS cells [26]. Thus, we speculated that miR-151-3p carried by CAF-EVs may activate the TGF-β signaling pathway by mediating the CHL1/integrin 1β axis, and then promote the growth and metastasis of OS.

To verify further this putative mechanism of oncogenesis, the MG63 cells were subjected to different treatments to elucidate the effect of miR-151-3p delivered

RETRACTED ARTICLE

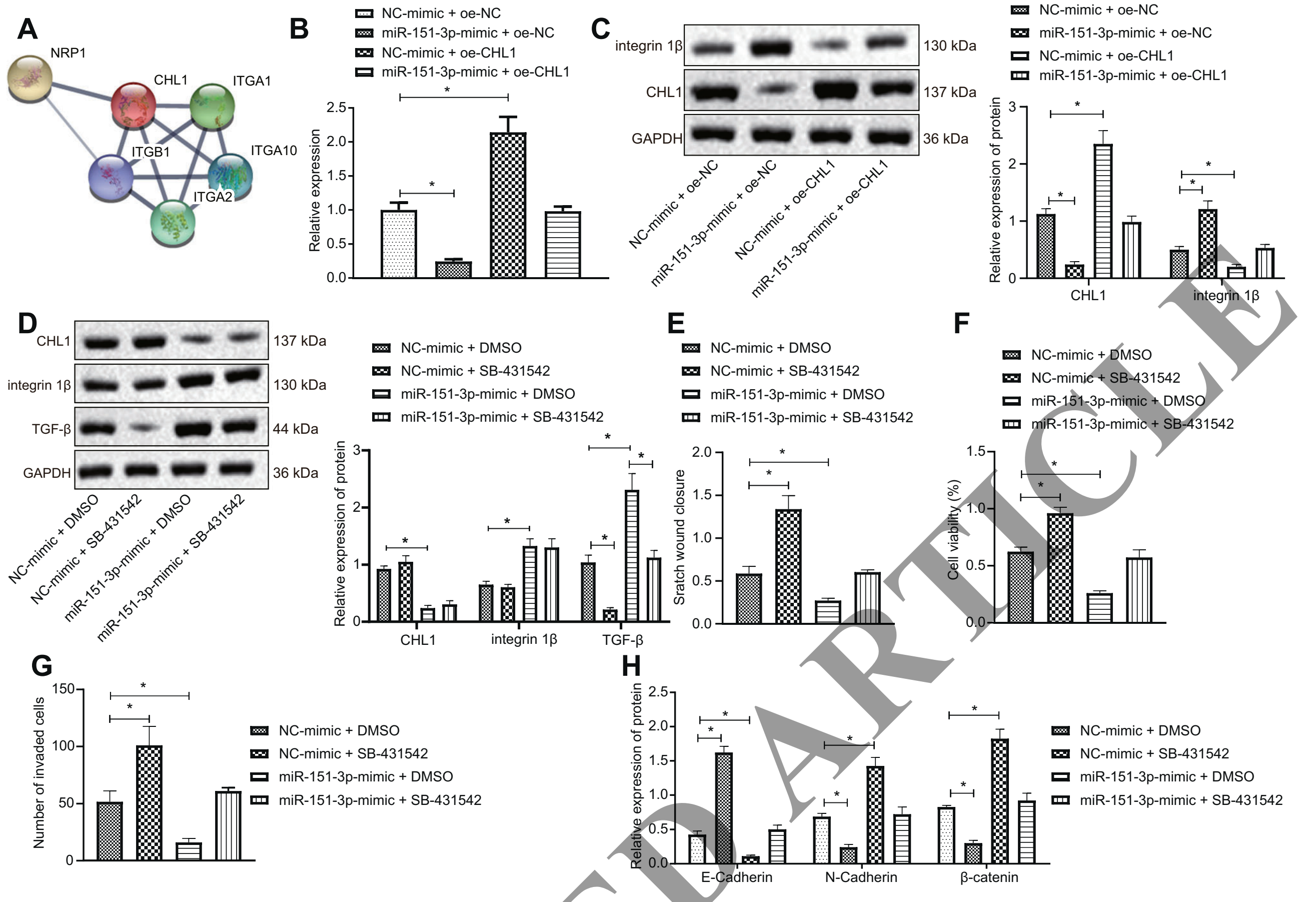

Fig. 5 MiR-151-3p regulates TGF-β via the CHL1/integrin1β axis in vitro to promote OS. **A** The interaction of gene network diagram of CHL1 in the GeneCards database. **B** qPCR detected the mRNA expression of CHL1 in MG63 cells transfected with miR-151-3p-mimic, oe-CHL1 or both. **C** Western blot analysis detected the expression of CHL1 and integrin 1β in MG63 cells transfected with miR-151-3p-mimic, oe-CHL1 or both, and normalized to GAPDH. **D** Western blot analysis detected the expression of CHL1, integrin 1β and TGF-β in MG63 cells treated with SB-431542, miR-151-3p-mimic, or both. **E** The migration of MG63 cells treated with SB-431542, miR-151-3p-mimic or both detected by wound healing experiment (200×). **F** The viability of MG63 cells treated with SB-431542, miR-151-3p-mimic, or both detected by CCK-8 assay. **G** The invasion of MG63 cells treated with SB-431542, miR-151-3p-mimic, or both detected by Transwell assay. **H** Western blot analysis detected expression of the EMT-related biomarkers E-Cadherin, β-catenin, and N-Cadherin in MG63 cells treated with SB-431542, miR-151-3p-mimic, or both. $*p < 0.05$, the data are measurement data reported as mean ± SD, which were analyzed by one-way ANOVA with Tukey's posthoc test. The experiment was repeated in triplicate.

RETRACTED ARTICLE

by CAF-EVs on growth and metastasis of OS. Then, qPCR was used to detect CHL1 mRNA expression with the results showing that compared to the NC-mimic + oe-NC group, CHL1 mRNA expression was increased in the NC-mimic + oe-CHL1 group while it was decreased in the miR-151-3p-mimic + oe-NC group. An inverted trend was evident in the miR-151-3p-mimic + oe-CHL1 group regarding the CHL1 mRNA expression as compared to the miR-151-3p-mimic + oe-NC group (Fig. 5B). In addition, Western blot analysis yielded similar results in the protein expression of CHL1 (Fig. 5C). Meanwhile, protein expression of integrin 1β was inhibited in the NC-mimic + oe-CHL1 group while an increase was noted in the miR-151-3p-mimic + oe-NC group compared to the NC-mimic + oe-NC group. Simultaneous overexpression of miR-151-3p and CHL1 decreased the integrin 1β protein expression.

For further validation, MG63 cells were treated with TGF-β pathway pharmacological inhibitor SB-431542 before performing the Western blot analysis. Compared to the NC-mimic + DMSO group, the protein expression of TGF-β was downregulated and that of CHL1 and integrin 1β showed no changes in the NC-mimic + SB-431542 group. However, when compared to the NC-mimic + DMSO group, in the miR-151-3p-mimic + DMSO group, CHL1 protein expression was reduced while TGF-β and integrin 1β protein expression was upregulated, all of which were reverted in the miR-151-3p-mimic + SB-431542 group (Fig. 5D).

Furthermore, experimental data from CCK-8, wound healing, and Transwell assays indicated that, compared to NC-mimic + DMSO group, the viability, migration, and invasion of MG63 cells were significantly decreased in the

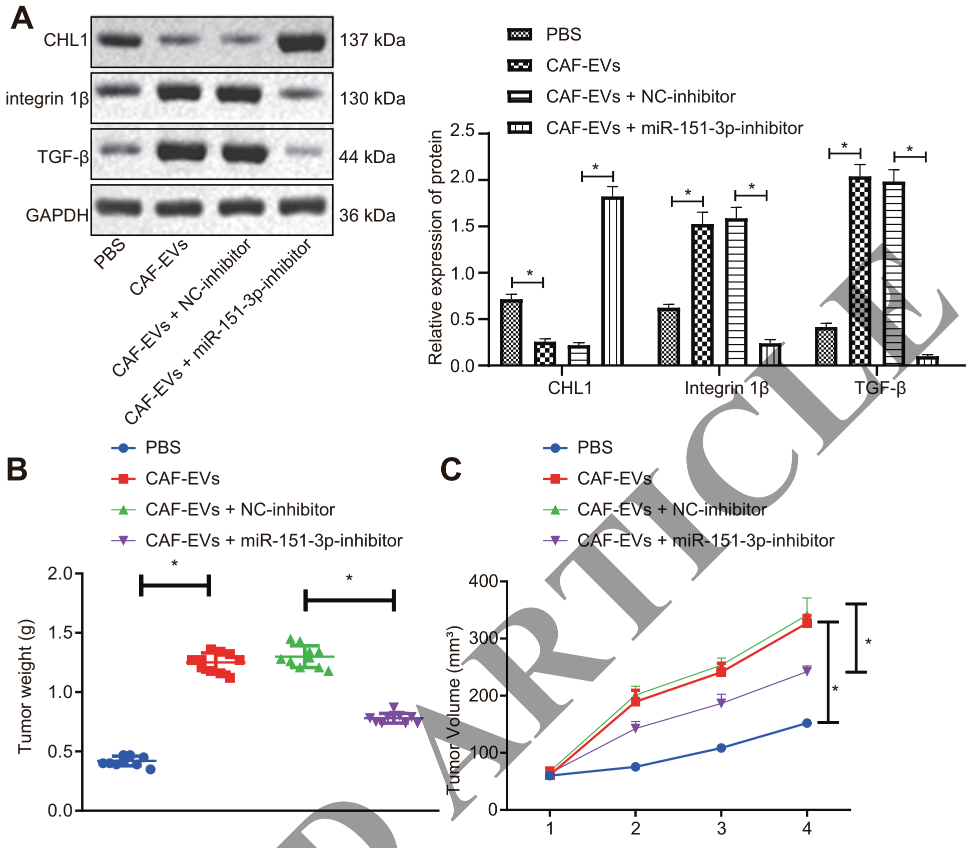

Fig. 6 miR-151-3p secretion of CAF-EVs promotes OS tumor growth in vivo. A Western blot analysis of CHL1, integer1β and TGFβ proteins in tumor tissues of nude mice treated with CAF-derived EVs or EVs from miR-151-3p-inhibitor-transfected CAFs. **B** Tumor weight of nude mice treated with CAF-derived EVs or EVs from miR-151-3p-inhibitor-transfected CAFs. **C** Tumor volume of nude mice treated with CAF-derived EVs or EVs from miR-151-3p-inhibitor-transfected CAFs. *$p < 0.05$. The data results were measurement data expressed as mean ± SD. Group comparisons were made by independent sample *t*-test analysis, comparison between multiple groups at multiple time points using repeated-measures ANOVA followed by Tukey's posthoc testing ($n = 10$).

NC-mimic + SB-431542 group while a contrary trend was evident in the miR-151-3p-mimic + DMSO group (Fig. 5E–G). On the other hand, Western blot analysis showed that, compared to NC-mimic + DMSO group, the expression of E-Cadherin was elevated while that of N-Cadherin and β-catenin was decreased in the NC-mimic + SB-431542 group. However, opposite results were observed in the miR-151-3p-mimic + DMSO group when compared with the NC-mimic + SB-431542 group (Fig. 5H). Collectively, these results suggested that the miR-151-3p delivered by CAF-EVs upregulated integrin 1β expression by targeting CHL1 and then activated the TGF-β pathway, thus promoting the proliferation, migration, invasion, and EMT of OS cells.

MiR-151-3p delivered by CAF-EVs promoted OS tumor growth in vivo

To verify further the pathological mechanism of CAF-EVs delivering miR-151-3p on OS in vivo, we established a mouse tumor model. The miR-151-3p-inhibitor and NC-inhibitor were transfected into CAF-EVs and then the transfected CAFs were injected intravenously into the mice, with PBS used as a control). Then, the OS tissue from the mice was isolated and the protein expression of CHL1, integer 1β, and TGFβ was detected by Western blot analysis. Contrary to findings in the PBS group, the expression of CHL1 in the CAF-EVs and CAF-EVs + NC-inhibitor groups was downregulated, while that of integrin1β and TGFβ was upregulated. Compared to the CAF-EVs + NC-inhibitor group, the expression of CHL1 was significantly up-regulated in the CAF-EVs + miR-151-3pinhibitor group, while that of integrin 1β and TGFβ was downregulated (Fig. 6A). Afterwards, the effects of miR-151-3p on mouse tumor formation were also detected, with determination of the tumor volume and weight (Fig. 6B–C). We found that, compared to the PBS group, the volume and weight of tumors were significantly increased in the CAF-EVs and CAF-EVs + NC-inhibitor groups. However, compared to the CAF-EVs-NC-inhibitor group, the volume and weight of tumors were significantly decreased in the EVs-miR-151-3p-inhibitor group. Hence, the above-mentioned results consistently showed that CAF-EVs delivering miR-151-3p facilitated the tumor growth of OS in vivo.

Discussion

Despite great research efforts to obtain better prognosis, the overall survival rate of patients with OS has remained dismal for decades [27]. Therefore, it is imperative to identify a

RETRACTED ARTICLE

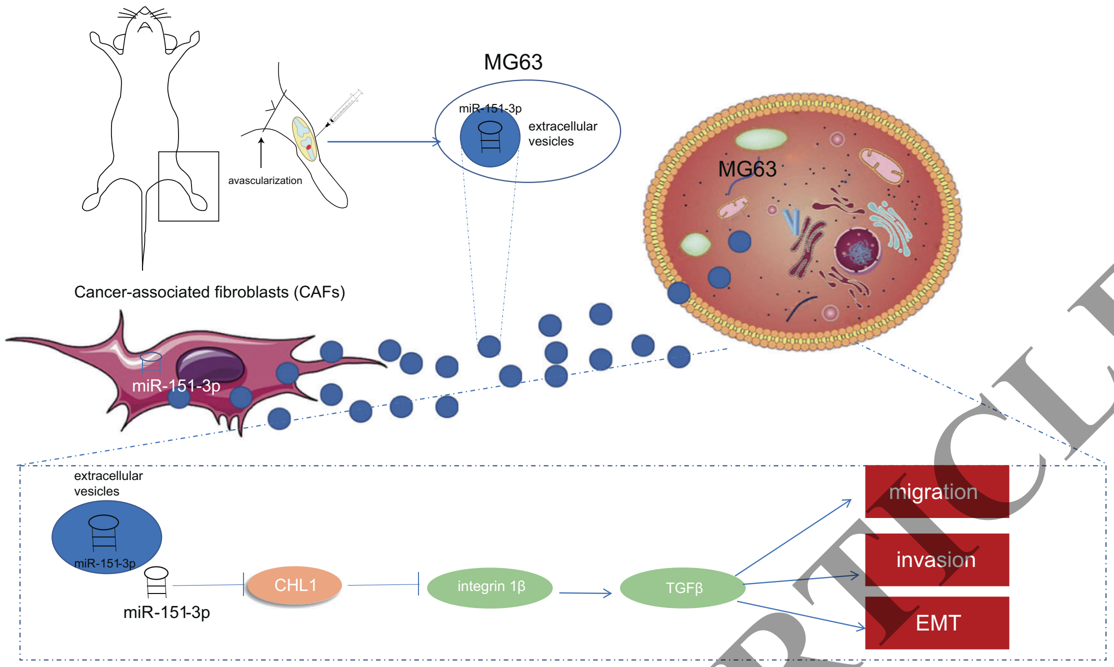

Fig. 7 The mechanism diagram. The transfer of miR-151-3p via CAF-EVs downregulates CHL1 and activates the TGF-β pathway via CHL1/integrin 1β axis to promote OS progression.

RETRACTED ARTICLE

novel effective therapeutic candidate for OS. Notably, CAFs and EVs-loaded miRNAs are of great importance to the tumor microenvironment, which is regarded as a determinant for cancer progression [28]. Correspondingly, in the present study, a series of gain-of-function and loss-of-function assays were performed to explore the CAF-derived EV communication in the tumor microenvironment of OS. Summarizing the present experimental data, we conclude that CAF-derived EVs promoted the acquisition of metastatic potential of cancer cells in OS by activating the TGF-β signaling pathway via the miR-151-3p-mediated CHL1/integrin 1β axis.

A fundamental observation made in our study was that miR-151-3p had high expression in OS and functioned as an oncogene. Intriguingly, in the context of breast cancer, miR-151 expression has been reported to be elevated while ectopically expressed miR-151 exhibited an inhibitory effect on breast cancer cell migration and invasion [14]. Moreover, high expression of miR-151-3p has been demonstrated in resected cholangiocarcinoma samples [15]. Furthermore, we now report that found miR-151-3p is highly expressed in EVs derived from CAFs, and contributes to the EMT process, migration, and invasion of OS cells. Nonetheless, CAFs have been recognized to be a driving force for colorectal cancer progression by releasing miRNA-loaded EVs [29]. Likewise, aggressive phenotypes of breast cancer cells have indicated to be significantly enhanced by treatment with EVs derived from CAFs containing multiple miRNAs [30, 31]. Largely in agreement with our present finding, a previous report has unraveled the tumor-promoting action of miR-1228 shuttled by EVs derived from CAFs in OS, and showed a mechanism of action via effects on the target gene, SCAI [13].

Correspondingly, our findings from the mechanistic investigation revealed CHL1 to be target gene of miR-151-3p, due to the presence of a specific binding site in CHL1 WT for miR-151-3p. Moreover, our data retrieved from the TCGA database on the UALCAN website indicated that CHL1 was expressed at a low level in OS samples. Concordantly, the downregulation of CHL1 has also been detected in colon adenocarcinoma, where it functioned as a direct target gene of miR-21-5p [32]. Intriguingly, a previously reported study has documented the tumor-suppressive property of CHL1 in esophageal squamous cell carcinoma by curbing proliferation and metastasis [23], suggesting an anti-tumor potential of CHL1 in OS. However, we have not yet validated the expression profile of CHL1 in clinically collected OS samples. We did subsequently explore the downstream signaling pathways possibly involved in the interaction between miR-151-3p and CHL1 in EVs released from CAFs, finding that CAFs-released EVs activated the TGF-β signaling pathway via the miR-151-3p-mediated CHL1/integrin 1β axis. Besides, CHL1 has been elaborated to suppress the growth and metastasis of nasopharyngeal carcinoma by interacting with integrin 1β [24]. Another study has indicated that the overexpression of integrin 1β promotes colorectal cancer cellular capabilities of proliferation, migration, and

invasion [22]. Moreover, in the context of non-small cell lung cancer, upregulated integrin 1β is considered as the hallmark of poor overall survival, while integrin 1β knockdown leads to the suppression of proliferative potential of cancer cells in vitro [33]. Accordingly, our study examined that integrin 1β expression was elevated in response to the delivery of miR-151-3p inhibitor, which gave rise to potentiated aggressive phenotypes of OS cells, suggesting that integrin 1β is worthy of future research in relation to OS. The TGF-β signaling pathway, which is conducive to the growth and metastasis of OS is reportedly activated by the integrin 1β [26, 34], providing further validation of our present results.

Taken together, we reported functional axis involving EV communication that is involved in the tumor microenvironment of OS. Herein, miR-151-3p loaded in EVs derived from CAFs possessed a pro-tumorigenic property through the target-inhibition of CHL1. Collectively, our study shows that CAFs can transfer miR-151-3p via EVs to OS cells, facilitating the EMT, migration, and invasion of OS cells both in vitro and in vivo (Fig. 7). Thus, we present novel insights into the pathogenesis of OS with identification of potential therapeutic targets to be tested in future studies.

Acknowledgements We would like to express our sincere appreciation to the reviewers for critical comments on this article.

Author contributions Ping Li and Gaoshan Qiao designed the study. Fangyong Hu, Leyin Zhu, and Cheng Xie collated the data, carried out data analyses, and produced the initial draft of the manuscript. Ping Li and Chengdong Zhu contributed to drafting the manuscript. Peng Wang, Changchao Wang, Xiaobo Tang, and Jian Wang contributed to the revision. All authors have read and approved the final submitted manuscript.

Compliance with ethical standards

Conflict of interest The authors declare that they have no conflict of interest.

Ethical statement All clinical studies were approved by the Ethics Committee of The People's Hospital of Yizheng City, The Affiliated Hospital of Yangzhou University, and conducted according to the guidelines of the Declaration of Helsinki. All OS and paracancerous tissue specimens were obtained from the oncology department of The People's Hospital of Yizheng City, The Affiliated Hospital of Yangzhou University. Patients signed informed consent for tissue donation in advance of their surgery.

Publisher's note Springer Nature remains neutral with regard to jurisdictional claims in published maps and institutional affiliations.

Open Access This article is licensed under a Creative Commons Attribution 4.0 International License, which permits use, sharing, adaptation, distribution and reproduction in any medium or format, as long as you give appropriate credit to the original author(s) and the source, provide a link to the Creative Commons license, and indicate if changes were made. The images or other third party material in this article are included in the article's Creative Commons license, unless indicated otherwise in a credit line to the material. If material is not included in the article's Creative Commons license and your intended use is not permitted by statutory regulation or exceeds the permitted use, you will need to obtain permission directly from the copyright holder. To view a copy of this license, visit http://creativecommons.org/licenses/by/4.0/.

References

1. Ritter J, Bielack SS. Osteosarcoma. Ann Oncol 2010;21: vii320–325.
2. Moore DD, Luu HH. Osteosarcoma. Cancer Treat Res 2014;162:65–92.
3. Harrison DJ, Geller DS, Gill JD, Lewis VO, Gorlick R. Current and future therapeutic approaches for osteosarcoma. Expert Rev Anticancer Ther 2018;18:39–50.
4. Shaikh AB, Li F, Li M, He B, He X, Chen G, et al. Present advances and future perspectives of molecular targeted therapy for osteosarcoma. Int J Mol Sci 2016;17:506.
5. Isakoff MS, Bielack SS, Meltzer P, Gorlick R. Osteosarcoma: current treatment and a collaborative pathway to success. J Clin Oncol 2015;33:3029–35.
6. Salvatore V, Teti G, Focaroli S, Mazzotti MC, Mazzotti A, Falconi M. The tumor microenvironment promotes cancer progression and cell migration. Oncotarget 2017;8:9608–16.
7. Bu L, Baba H, Yoshida N, Miyake K, Yasuda T, Uchihara T, et al. Biological heterogeneity and versatility of cancer-associated fibroblasts in the tumor microenvironment. Oncogene 2019;38: 4887–901.
8. Yang F, Ning Z, Ma L, Liu W, Shao C, Shu Y, et al. Exosomal miRNAs and miRNA dysregulation in cancer-associated fibroblasts. Mol Cancer 2017;16:148.
9. Azmi AS, Bao B, Sarkar FH. Exosomes in cancer development, metastasis, and drug resistance: a comprehensive review. Cancer Metastasis Rev 2013;32:623–42.
10. Kalluri R, LeBleu VS. The biology, function, and biomedical applications of exosomes. Science 2020;367:6478.
11. Yu X, Odenthal M, Fries JW. Exosomes as miRNA carriers: formation-function-future. Int J Mol Sci. 2016;17:2028.
12. Gilligan KE, Dwyer RM. Engineering exosomes for cancer therapy. Int J Mol Sci. 2017;18:1122
13. Wang JW, Wu XF, Gu XJ, Jiang XH. Exosomal miR-1228 from cancer-associated fibroblasts promotes cell migration and invasion of osteosarcoma by directly targeting SCAI. Oncol Res. 2019;27:979–86.
14. Yeh TC, Huang TT, Yeh TS, Chen YR, Hsu KW, Yin PH, et al. miR-151-3p targets TWIST1 to repress migration of human breast cancer cells. PLoS ONE 2016;11:e0168171.
15. McNally ME, Collins A, Wojcik SE, Liu J, Henry JC, Jiang J, et al. Concomitant dysregulation of microRNAs miR-151-3p and miR-126 correlates with improved survival in resected cholangiocarcinoma. HPB 2013;15:260–4.
16. Oved K, Morag A, Pasmanik-Chor M, Oron-Karni V, Shomron N, Rehavi M, et al. Genome-wide miRNA expression profiling of human lymphoblastoid cell lines identifies tentative SSRI antidepressant response biomarkers. Pharmacogenomics 2012;13: 1129–39.
17. Qin X, Guo H, Wang X, Zhu X, Yan M, Wang X, et al. Exosomal miR-196a derived from cancer-associated fibroblasts confers cisplatin resistance in head and neck cancer through targeting CDKN1B and ING5. Genome Biol 2019;20:12.
18. Thery C, Witwer KW, Aikawa E, Alcaraz MJ, Anderson JD, Andriantsitohaina R, et al. Minimal information for studies of

RETRACTED ARTICLE

extracellular vesicles 2018 (MISEV2018): a position statement of the International Society for Extracellular Vesicles and update of the MISEV2014 guidelines. J Extracell Vesicles 2018;7:1535750.
19. Dourado MR, Korvala J, Astrom P, De Oliveira CE, Cervigne NK, Mofatto LS, et al. Extracellular vesicles derived from cancer-associated fibroblasts induce the migration and invasion of oral squamous cell carcinoma. J Extracell Vesicles 2019;8:1578525.
20. Zhang Z, Li X, Sun W, Yue S, Yang J, Li J, et al. Loss of exosomal miR-320a from cancer-associated fibroblasts contributes to HCC proliferation and metastasis. Cancer Lett 2017;397:33–42.
21. Raimondi L, De Luca A, Gallo A, Costa V, Russelli G, Cuscino N, et al. Osteosarcoma cell-derived exosomes affect tumor microenvironment by specific packaging of microRNAs. Carcinogenesis. 2019;41: 666–77.
22. Ha YJ, Tak KH, Kim SK, Kim CW, Lee JL, Roh SA, et al. Biological characteristics and clinical significance of ITGB1 and RHOC in patients with recurrent colorectal cancer. Anticancer Res 2019;39:4853–64.
23. Tang H, Jiang L, Zhu C, Liu R, Wu Y, Yan Q, et al. Loss of cell adhesion molecule L1 like promotes tumor growth and metastasis in esophageal squamous cell carcinoma. Oncogene 2019;38:3119–33.
24. Chen J, Jiang C, Fu L, Zhu CL, Xiang YQ, Jiang LX, et al. CHL1 suppresses tumor growth and metastasis in nasopharyngeal carcinoma by repressing PI3K/AKT signaling pathway via interaction with Integrin beta1 and Merlin. Int J Biol Sci 2019;15:1802–15.
25. Chen CA, Chang JM, Chang EE, Chen HC, Yang YL. TGF-beta1 modulates podocyte migration by regulating the expression of integrin-beta1 and -beta3 through different signaling pathways. Biomed Pharmacother 2018;105:974–80.
26. Xu X, Liu M. miR-522 stimulates TGF-beta/Smad signaling pathway and promotes osteosarcoma tumorigenesis by targeting PPM1A. J Cell Biochem 2019;120:18425–34.
27. Bishop MW, Janeway KA, Gorlick R. Future directions in the treatment of osteosarcoma. Curr Opin Pediatr 2016;28:26–33.
28. Eichelmann AK, Matuszcak C, Hummel R, Haier J. Role of miRNAs in cell signaling of cancer associated fibroblasts. Int J Biochem Cell Biol 2018;101:94–102.
29. Bhome R, Goh RW, Bullock MD, Pillar N, Thirdborough SM, Mellone M, et al. Exosomal microRNAs derived from colorectal cancer-associated fibroblasts: role in driving cancer progression. Aging 2017;9:2666–94.
30. Donnarumma E, Fiore D, Nappa M, Roscigno G, Adamo A, Iaboni M, et al. Cancer-associated fibroblasts release exosomal microRNAs that dictate an aggressive phenotype in breast cancer. Oncotarget 2017;8:19592–608.
31. Wang H. et al. MicroRNA-181d-5p-containing exosomes derived from CAFs promote EMT by regulating CDX2/HOXA5 in breast cancer. Mol Ther Nucleic Acids. 2020;19:654–67.
32. Yu W, Zhu K, Wang Y, Yu H, Guo J. Overexpression of miR-21-5p promotes proliferation and invasion of colon adenocarcinoma cells through targeting CHL1. Mol Med 2018;24:36.
33. Liang Z, Kong R, He Z, Lin LY, Qin SS, Chen CY, et al. High expression of miR-493-5p positively correlates with clinical prognosis of non small cell lung cancer by targeting oncogene ITGB1. Oncotarget 2017;8:47389–99.
34. Asai K, Funaba M, Murakami M. Enhancement of RANKL-induced MITF-E expression and osteoclastogenesis by TGF-beta. Cell Biochem Funct 2014;32:401–9.

RETRACTED ARTICLE
